# A Common Pathway for Activation of Host-Targeting and Bacteria-Targeting Toxins in Human Intestinal Bacteria

**DOI:** 10.1128/mBio.00656-21

**Published:** 2021-08-31

**Authors:** Yiqiao Bao, Andrew A. Verdegaal, Brent W. Anderson, Natasha A. Barry, Jing He, Xiang Gao, Andrew L. Goodman

**Affiliations:** a Department of Microbial Pathogenesis, Yale University, New Haven, Connecticut, USA; b Microbial Sciences Institute, Yale University, New Haven, Connecticut, USA; c State Key Laboratory of Microbial Technology, Shandong University, Qingdao, Shandong, China; University of Connecticut

**Keywords:** bacteriocin, *Bacteroides*, interbacterial interaction, microbiome

## Abstract

Human gut microbes exhibit a spectrum of cooperative and antagonistic interactions with their host and also with other microbes. The major *Bacteroides* host-targeting virulence factor, Bacteroides fragilis toxin (BFT), is produced as an inactive protoxin by enterotoxigenic B. fragilis strains. BFT is processed by the conserved bacterial cysteine protease fragipain (Fpn), which is also encoded in B. fragilis strains that lack BFT. In this report, we identify a secreted antibacterial protein (fragipain-activated bacteriocin 1 [Fab1]) and its cognate immunity protein (resistance to fragipain-activated bacteriocin 1 [RFab1]) in enterotoxigenic and nontoxigenic strains of B. fragilis. Although BFT and Fab1 share no sequence identity, Fpn also activates the Fab1 protoxin, resulting in its secretion and antibacterial activity. These findings highlight commonalities between host- and bacterium-targeting toxins in intestinal bacteria and suggest that antibacterial antagonism may promote the conservation of pathways that activate host-targeting virulence factors.

## INTRODUCTION

The human intestine harbors a complex microbial community that inhabits the length of the gastrointestinal tract, with densities being highest in the colon. In most individuals, the gut microbiome is dominated by representatives of two major phyla (*Bacteroidetes* and *Firmicutes*). Although these broad taxonomic groups are ubiquitous across individuals, species- and strain-level differences within these phyla are associated with differences in pathogen susceptibility, metabolism, drug response, and other host phenotypes (1). Notably, gut microbial strains can persist in individuals for years or decades despite continual challenges from the outside environment (2). Multiple mechanisms likely contribute to strain persistence or replacement, including priority effects (3) and nutrient specialization (4). In addition, interbacterial antagonism is increasingly recognized as a factor that determines strain selection and competition in this densely packed ecosystem (5). Antagonistic mechanisms allow bacteria to selectively target closely related and/or physically proximal cells and leave characteristic signatures in gut microbial genomes and metagenomes (6, 7). Understanding these antagonistic interactions can provide insight into the rules of assembly in the gut microbiome and inform future therapeutic manipulation of these microbial communities.

Genetic screens, biochemical isolations, and bioinformatic approaches have identified antimicrobial toxins produced by a wide variety of intestinal bacteria, including *Lactobacillus*, *Bifidobacterium*, *Enterococcus*, *Bacteroides*, and Escherichia. These studies highlight two general antagonistic strategies: contact-dependent mechanisms include contact-dependent inhibition (CDI) and the type VI secretion system (T6SS) (8, 9); contact-independent antibacterial factors include small-molecule antibiotics and secreted antimicrobial peptides and proteins (e.g., microcins and colicins) (10–13). These antimicrobial factors can mediate competition between bacterial cells across families (broad spectrum) or within strains of the same species (narrow spectrum) (5, 13). Contact-dependent and contact-independent antibacterial factors use a variety of mechanisms of action, including pore formation and inhibition of DNA, RNA, or protein synthesis (13). Both contact-dependent and diffusible toxins can limit the expansion of competing commensals and pathogens *in vivo* (10, 14, 15).

These systems have been best studied in *Proteobacteria*; identification and characterization of antimicrobial factors in human gut *Bacteroides* is constrained by the absence of sequence similarity or protein motifs from previously studied antibacterial effectors. While T6SS-delivered effectors can be identified by genomic context (16), the factors that mediate contact-independent antagonism in human gut *Bacteroides* have been elusive. Genetic approaches have identified broad-spectrum peptide toxins that target diverse members of the phylum *Bacteroidetes* (bacteroidetocin A and bacteroidetocin B) (17) and larger proteins that specifically target strains within the same species (18–21). These narrow-spectrum antimicrobial factors share eukaryotic-like features, including membrane attack complex/perforin (MACPF) or ubiquitin-like domains (18–21).

Bacteroides fragilis encodes a diverse repertoire of T6SS-dependent effectors and contact-independent bacteriocins (5). Notably, B. fragilis is implicated in both health and disease (22). This species produces beneficial immunomodulatory factors that mediate host immune system development (23) but can also cause epithelial cell damage, making it the most common anaerobic isolate from abdominal abscesses and bloodstream infections (22). Enterotoxigenic B. fragilis (ETBF) strains are marked by the presence of pathogenicity islands that encode B. fragilis toxin (BFT), which cleaves E-cadherin and causes colonic cell damage and inflammation (24). Fragipain (Fpn), a cysteine protease encoded outside the pathogenicity islands, transforms the 45-kDa full-length BFT protoxin into its 20-kDa active form through cleavage at an arginine-alanine site (25, 26). Interestingly, BFT from an *fpn* mutant strain is readily activated by host proteases in the gut and efficiently causes epithelial cell damage (25). Fpn is also conserved in nontoxigenic B. fragilis (NTBF) strains that lack BFT, suggesting other roles for Fpn beyond activation of this host-targeting toxin (25, 26). Consistent with this observation, a recent report identifies numerous differences between the secretomes of a wild-type ETBF strain and its isogenic *fpn* mutant (26).

Here, we report that many NTBF and ETBF B. fragilis strains use Fpn for activation and secretion of a potent, secreted antibacterial toxin. This antibacterial protein, fragipain-activated bacteriocin 1 (Fab1), lacks domains found in previously characterized *Bacteroides* bacteriocins and directly kills susceptible strains upon activation by Fpn. Transfer of the open reading frame downstream of *fab1* into otherwise susceptible strains confers protection from Fab1, suggesting that this downstream gene encodes an immunity protein. Together, these results expand the repertoire of antagonistic activities in human gut microbes, suggest that contact-independent host- and bacteria-targeting toxins can leverage the same machinery for activation and provide an explanation for the maintenance of this machinery in the absence of its best-characterized substrate.

## RESULTS

### Bacteroides fragilis exhibits potent antibacterial activity independent of type VI secretion.

**FIG 1:**
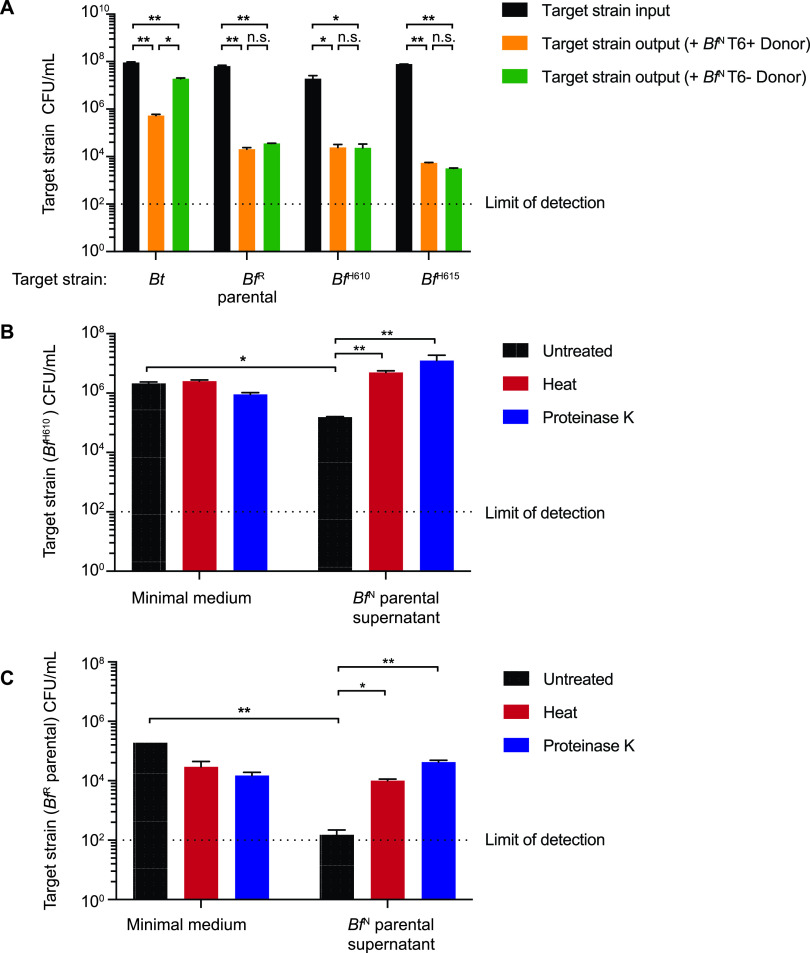
B. fragilis strain NCTC9343 (*Bf*^N^) secretes antimicrobial protein(s) to target susceptible B. fragilis strains independent of the type VI secretion system. (A). Input and output CFU of target strains after surface cocultivation with the indicated *Bf*^N^ producer strains. Error bars indicate standard deviations (SD) (*n* = 2; representative of three independent experiments). Strain designations: *Bt*, B. thetaiotaomicron*; Bf*^R^, B. fragilis strain 638R; *Bf*^H610^, B. fragilis strain HMW610; *Bf*^H615^, B. fragilis strain HMW615. Parental strains carry deletions in *tssC* which disable the type VI secretion system (T6SS). (B and C). *Bf*^N^ culture supernatants contain antimicrobial activity. CFU of two target strains after exposure to culture supernatant from the *Bf*^N^ parental strain or minimal medium with different treatments are reported. Error bars indicate SD (*n* = 2; representative of three independent experiments). *, *P < *0.05; **, *P < *0.01; n.s., not significant.

In the course of screening B. fragilis isolates for contact-dependent antibacterial activity, we observed that the T6SS-positive NTBF strain NCTC9343 (*Bf*^N^) exhibits potent antagonistic activity toward diverse B. fragilis strains that is independent of a functional T6SS (Fig. 1A). Target strains (B. fragilis HMW160 [*Bf*^H610^], B. fragilis HMW615 [*Bf*^H615^], and B. fragilis 638R [*Bf*^R^]) were selected to represent distinct branches of the B. fragilis phylogeny (14) that can be differentiated from *Bf*^N^ by selective plating (see Table S1 in the supplemental material). By contrast, killing of Bacteroides thetaiotaomicron by *Bf*^N^ was T6SS dependent, as previously reported (14). To distinguish this T6SS-independent activity from T6SS-dependent antagonism, we used deletion mutants in the essential T6SS component *tssC* (referred to as parental strains) in subsequent experiments. Culture supernatants from the *Bf*^N^ parental strain also possess bactericidal activity toward susceptible B. fragilis strains (Fig. 1B and C) but not B. thetaiotaomicron (Fig. S1). Both heat and proteinase K treatment abolished this T6SS-independent antimicrobial activity, suggesting that one or more secreted protein factor(s) are required (Fig. 1B and C). Notably, *Bf*^N^ lacks homologs of previously reported MACPF (membrane attack complex/perforin) domain-containing antimicrobial proteins and bacteroidetocin peptide toxins (17–19, 21, 27).

10.1128/mBio.00656-21.1 FIG S1 B. thetaiotaomicron (*Bt*) is resistant to the antimicrobial protein(s) produced by *Bf*^N^. B. thetaiotaomicron CFU after exposure to *Bf*^N^ parental culture supernatant or minimal medium with different treatments are reported. Error bars indicate SD (*n* = 2; representative of three independent experiments). Download FIG S1, EPS file, 1.9 MB. Copyright © 2021 Bao et al. 2021 Bao et al. https://creativecommons.org/licenses/by/4.0/ This content is distributed under the terms of the Creative Commons Attribution 4.0 International license.

10.1128/mBio.00656-21.6 TABLE S1 Bacterial strains, plasmids, and oligonucleotide primers used in this study. Download Table S1, XLSX file, 0.02 MB. Copyright © 2021 Bao et al. 2021 Bao et al. https://creativecommons.org/licenses/by/4.0/ This content is distributed under the terms of the Creative Commons Attribution 4.0 International license.

### Identification of genetic regions required for *Bf*^N^ antagonistic activity.

**FIG 2:**
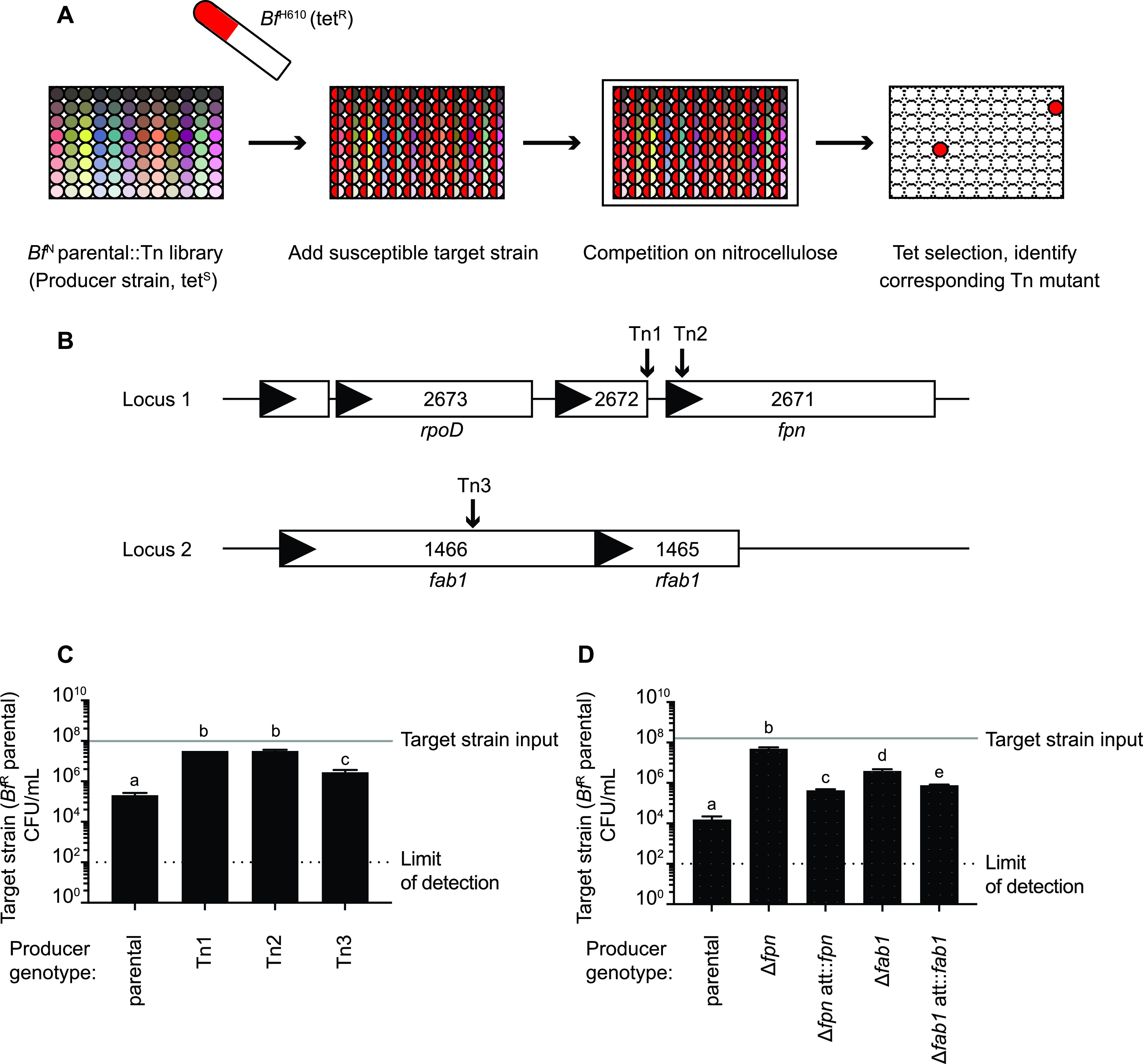
A loss-of-function (LoF) genetic screen identifies genes involved in antimicrobial activity. (A) Workflow of the LoF genetic screen. (B) Genomic locations of *Bf*^N^ transposon insertions that abrogate its capacity to inhibit growth of *Bf*^H610^. (C) Quantification of killing activity by *Bf*^N^ parental and transposon mutant strains identified in panel B. (D) Quantification of killing activity by *Bf*^N^ parental, isogenic deletion strains, and complemented mutants. Complemented mutants carry the deleted gene in single copy in a neutral locus (*att*). For panels C and D, mean number of target strain input CFU is indicated with a gray line (mean = 2.2 × 10^7^); line width represents SD (SD = 8.5 × 10^6^). Target strain output CFU are reported. Error bars indicate SD (*n* = 2; representative of three independent experiments). Different letters represent groups that are statistically significantly different (*P < *0.05).

To identify genetic regions required for antibacterial activity, we constructed a *mariner* transposon mutant library in the *Bf*^N^ parental background and screened individual clones for loss of antagonism toward *Bf*^H610^. This representative target strain is susceptible to the T6SS-independent *Bf*^N^ antagonistic activity (Fig. 1A and B) and, unlike *Bf*^N^, is naturally resistant to tetracycline. As a result, selective plating of the competition assay on tetracycline serves as an indicator of the extent of *Bf*^N^::TN antagonistic activity (Fig. 2A). From a total of 15,000 mutants screened, three independent clones (carrying transposon insertions in the intergenic region upstream of BF9343_2671, within the BF9343_2671 open reading frame, and within the BF9343_1466 open reading frame) demonstrated significant reductions in bactericidal activity (Fig. 2B). Based on results described below, we designated BF9343_2671 as *fpn* and BF9343_1466 as *fab1.* The strain carrying a transposon in the intergenic region upstream of *fpn* exhibited significantly reduced *fpn* expression (Fig. S2). For subsequent studies, we used the tetracycline-sensitive, genetically tractable NTBF parent strain 638R (*Bf*^R^) strain as a representative target strain. Unmarked, in-frame deletions of *fpn* and *fab1* in *Bf*^N^ recapitulate the decreased antibacterial capacity of the transposon mutant strains, and complementation of gene expression in single copy in *trans* significantly (but not completely) restores antagonistic activity (Fig. 2C and D). Together, these results implicate *fpn* and *fab1* in *Bf*^N^ antagonistic activity.

10.1128/mBio.00656-21.2 FIG S2 Transposon insertion in the intergenic region between *BF9343_2672* and *fpn* significantly reduces *fpn* gene expression. Expression of *fpn* and *BF9343_2672* in the mutant strain carrying a transposon insertion in the intergenic region upstream of *fpn* (Tn1) (Fig. 2B) relative to the *Bf*^N^ parental strain was determined by reverse transcription-qPCR (qRT-PCR). Mean expression levels are calculated from technical triplicates from two independent experiments and normalized to 16S RNA. Error bars indicate SD. Download FIG S2, EPS file, 1.9 MB. Copyright © 2021 Bao et al. 2021 Bao et al. https://creativecommons.org/licenses/by/4.0/ This content is distributed under the terms of the Creative Commons Attribution 4.0 International license.

### Designation of BF9343_2671 as *fpn*.

**FIG 3:**
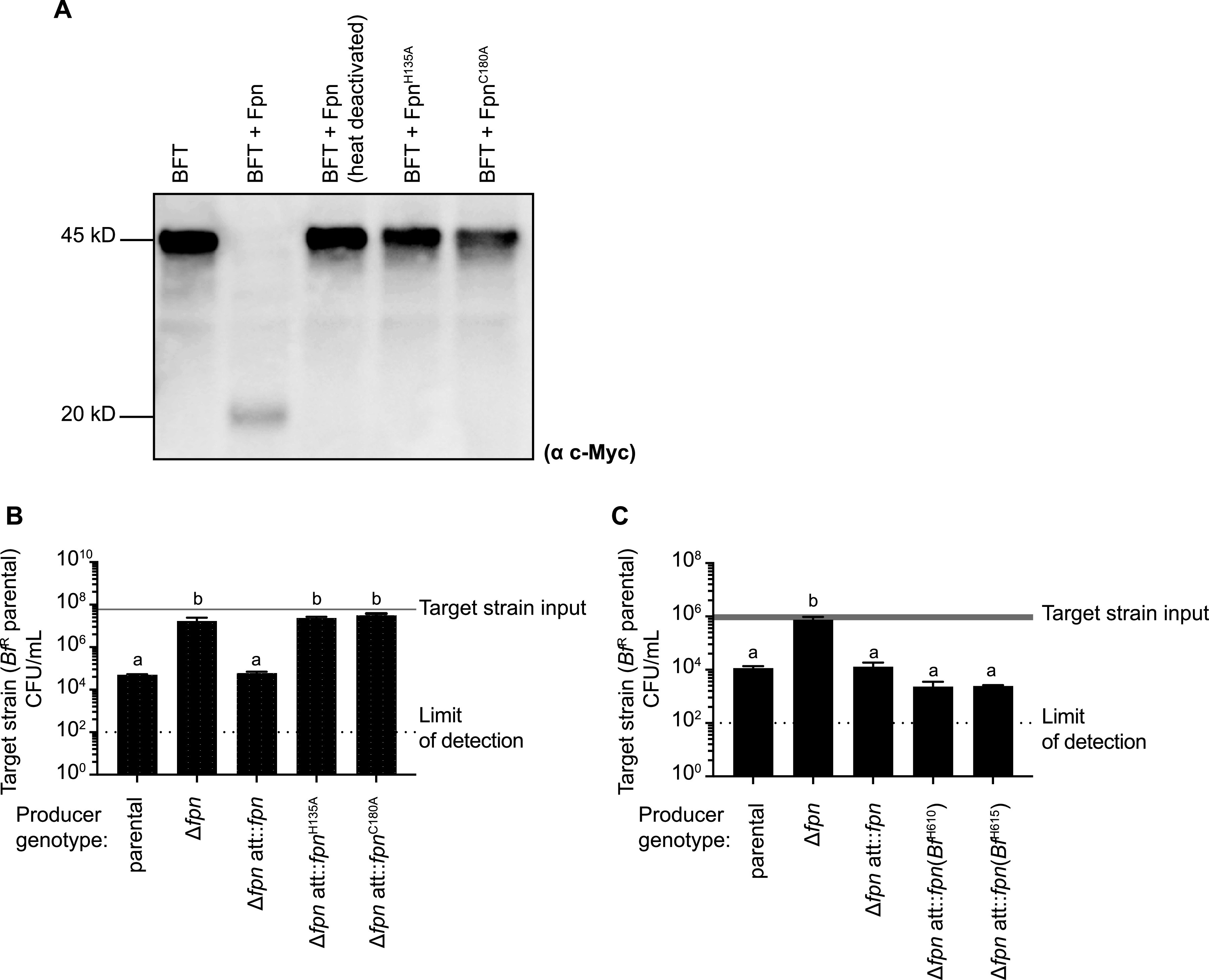
Activity of Fpn homologs from *Bf*^N^ and susceptible target strains. (A) Western blot analysis of BFT products generated following incubation of purified *Bf*^N^ Fpn or its catalytic residue mutants (Fpn^H135A^; Fpn^C180A^) with BFT carrying a C-terminal c-Myc tag. (B) Mutation of Fpn catalytic residues in *Bf*^N^ abrogates its ability to inhibit *Bf*^R^ growth. The mean number of target strain input CFU is indicated with a gray line (mean = 6.0 × 10^7^); line width represents SD (SD = 5.0 × 10^6^). Target strain output CFU are reported. Error bars indicate SD (*n* = 2; representative of three independent experiments). (C) Expression of *fpn* genes derived from susceptible strains *Bf*^H610^ and *Bf*^H615^ restores killing activity to a *Bf*^N^Δ*fpn* mutant. The mean number of target strain input CFU is indicated with a gray line (mean = 9.6 × 10^5^); line width represents SD (SD = 2.4 × 10^5^). Target strain output CFU are reported. Error bars indicate SD (*n* = 2; representative of three independent experiments). For panels B and C, different letters represent groups that are statistically significantly different (*P < *0.05).

*Bf*^N^ Fpn shares 99.7% identity with a clostripain-related cysteine protease that mediates maturation of the host-targeting toxin BFT in ETBF strains (25). This activity requires a conserved histidine-cysteine dyad, which is common to cysteine proteases (28). We expressed and purified *Bf*^N^ Fpn in Escherichia coli. Although *Bf*^N^ does not encode BFT, this purified Fpn also cleaves purified BFT to form the 20-kDa toxin (Fig. 3A). Cleavage activity is abrogated by heat deactivation of Fpn or by substitution of the predicted active site residues H^135^ or C^180^ with alanine (Fig. 3A). The active-site residues required for the BFT cleavage role of Fpn are also required for its contribution to antimicrobial activity (Fig. 3B), suggesting that the cysteine protease activity of this protein is also important for its contribution to antimicrobial antagonism.

Surprisingly, *Bf*^H610^ and *Bf*^H615^, which are both susceptible to *Bf^N^* antimicrobial activity (Fig. 1A), also encode Fpn homologs. Expression of each homolog restores antimicrobial activity to the *Bf*^N^ Δ*fpn* deletion strain, indicating that Fpn homologs from these susceptible B. fragilis strains are functional (Fig. 3C). This result also suggests that the antimicrobial capacity of *Bf*^N^ is dependent on Fpn and another factor that is missing in *Bf*^H610^ and *Bf*^H615^.

### Fpn activates the antimicrobial function of Fab1.

**FIG 4:**
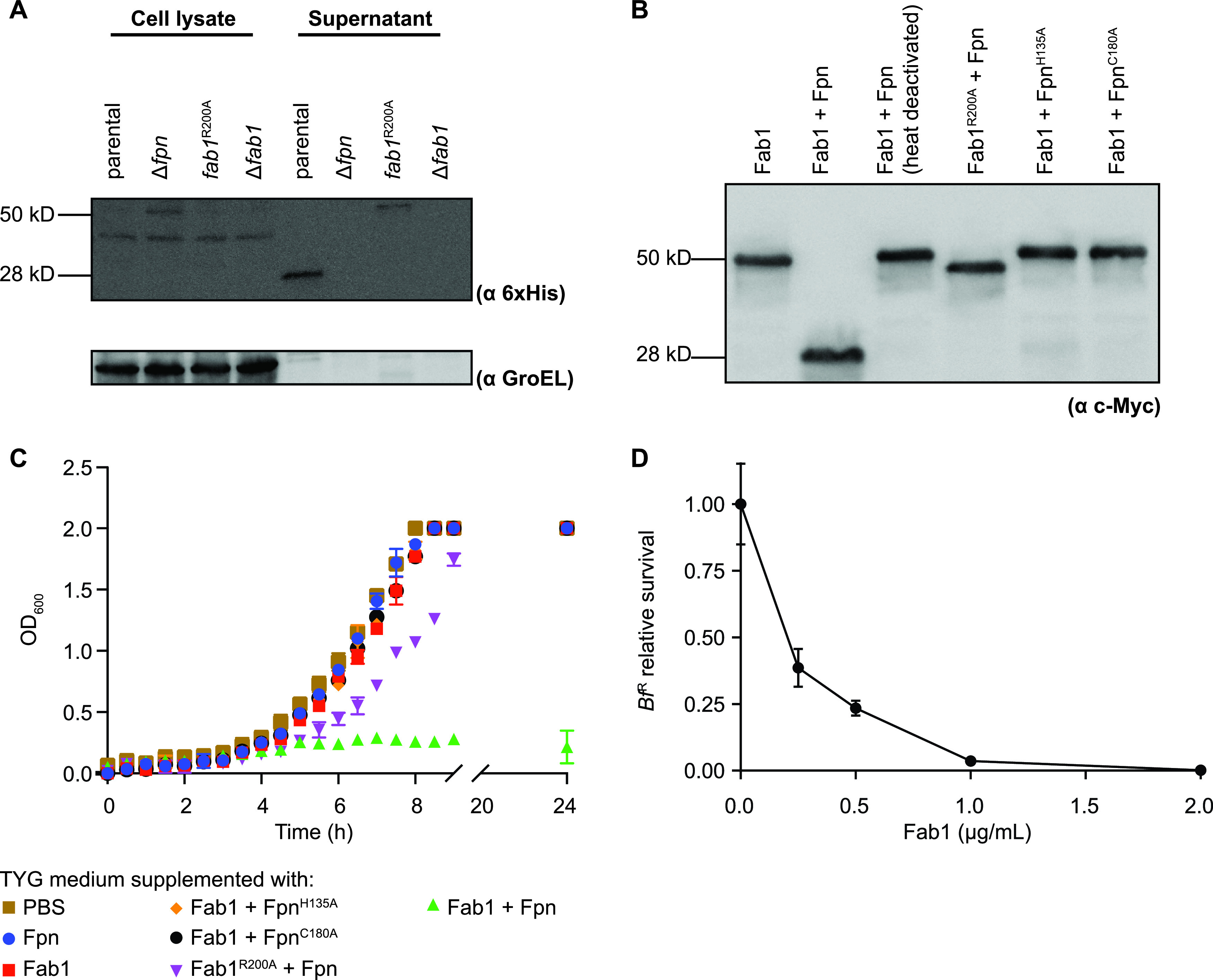
Fpn cleaves Fab1 to activate its antimicrobial function. (A) Western blot analysis of *Bf*^N^ parental and mutant strains carrying Fab1-His_6_ in the native genomic location and probed with anti-6×His. (B) Western blot analysis of Fab1 products generated following incubation of purified Fpn (wild type or catalytic residue mutants) with purified Fab1 (wild type or cleavage site mutant). Fab1 carries a C-terminal c-Myc tag; Western blots were probed with anti-c-Myc. (C) Growth of the susceptible *Bf*^R^ parental strain in TYG medium supplemented with combinations of purified Fpn (wild type or catalytic residue mutants) and purified Fab1 (wild type or cleavage site mutant). Error bars indicate SD (*n* = 2; representative of three independent experiments). (D) Concentration dependence of Fab1 activity on *Bf*^R^ viability. Fpn is included at 2 μg/ml under all conditions. Cell viability (CFU/ml) is normalized to the mean viability of cultures incubated in the absence of Fab1. Error bars indicate SD (*n* = 2; representative of three independent experiments).

The loss-of-function screen also implicated Fab1, a predicted 50-kDa protein with no known function or recognizable domains, in *Bf*^N^ antimicrobial activity (Fig. 2B). Fab1 homologs are absent in *Bf*^H610^ and *Bf*^H615^. C-terminal epitope tagging of the Fab1 open reading frame in *Bf*^N^ followed by Western blotting using an epitope-targeted antibody revealed that the C-terminal end of the protein is almost entirely localized to the secreted (supernatant) fraction as a 28-kDa fragment (Fig. 4A). Untargeted secretome analysis also suggests that Fab1 is abundant in culture supernatants (14). Because both Fpn and Fab1 are required for antimicrobial activity, we next examined the impact of Fpn on the production and secretion of the 28-kDa C-terminal fragment of Fab1. Indeed, the Δ*fpn* deletion strain fails to secrete this 28-kDa fragment of Fab1 and instead accumulates the full-length (50-kDa) protein in the cell pellet (Fig. 4A). To determine whether Fpn acts directly on Fab1, we next incubated purified Fab1 with purified Fpn and examined Fab1 processing by Western blotting. Indeed, Fpn directly cleaves recombinant Fab1 to produce a 28-kDa C-terminal fragment (as observed in the supernatant of the *Bf*^N^ parental strain but not the *Bf*^N^ Δ*fpn* mutant); heat deactivation of Fpn or mutating its active site residues abolishes this activity (Fig. 4B). Using liquid chromatography-mass spectrometry (LC-MS), we identified a cleavage site between amino acid residues R200 and A201 of Fab1 that produces the secreted 28-kDa fragment (Fig. S3). Consistent with this prediction, Fab1^R200A^ is not processed into the 28-kDa form upon incubation with Fpn *in vivo* (Fig. 4A) or *in vitro* (Fig. 4B). This arginine-alanine sequence in Fab1 is consistent with the primary cleavage site of BFT (R211-A212) targeted by Fpn (25). Fab1 likely contains an additional cleavage site(s), because Fab1^R200A^ is secreted (Fig. 4A) and migrates at a different size than Fab1 controls in which Fpn is absent (Fig. 4B).

10.1128/mBio.00656-21.3 FIG S3 Fpn cleaves Fab1 between R200 and A201 to produce the secreted 28-kDa fragment. (A) Fab1 peptides identified by liquid chromatography-mass spectrometry (LC-MS). (B) Comparison of the number of peptide spectrum matches (PSMs) between full-length Fab1 and its Fpn-dependent C-terminal 28-kDa fragment shows that the primary cleavage site of Fab1 is between R200 and A201. Peptide locations A to J are as described in panel A. Download FIG S3, EPS file, 1.7 MB. Copyright © 2021 Bao et al. 2021 Bao et al. https://creativecommons.org/licenses/by/4.0/ This content is distributed under the terms of the Creative Commons Attribution 4.0 International license.

To assess whether Fpn, full-length Fab1, or the Fpn-processed Fab1 is responsible for the observed antibacterial activity, we next grew the susceptible *Bf*^R^ parental strain in the presence of these proteins, either alone or in combination, and determined growth rates of *Bf*^R^. While neither Fpn nor Fab1 alone inhibited growth, the combination of these proteins exhibited potent antibacterial activity (Fig. 4C). Substitution of Fpn with catalytically inactive mutants or substitution of Fab1 with Fab1^R200A^ blocked toxicity, indicating that processing of Fab1 is required for its antibacterial activity (Fig. 4C). Titrating purified Fab1 indicates an MIC_50_ of 0.2 μg/ml under these conditions (Fig. 4D). Together, these results suggest that Fab1 is an antibacterial protoxin that is processed by Fpn into a C-terminal 28-kDa form to mediate its secretion and activity. Based on these results, we designated this protein fragipain-activated bacteriocin 1 (Fab1).

Gut microbes use antibacterial antagonism to prevent or delay expansion of invading strains (5). In the genus *Bacteroides*, invasion is also influenced by the origin of the invading strain: bacterial cultures prepared *in vitro* have a diminished invasion capacity compared to *in vivo-*prepared bacteria collected from the feces of monocolonized gnotobiotic mice (3). To examine the role of Fab1 in strain dynamics in the gut environment, we colonized germfree Swiss-Webster (outbred) mice with either the *Bf*^N^ parental or *Bf*^N^Δ*fab1* strains and measured the ability of *in vivo*-prepared *Bf*^R^ parental cells to invade the gastrointestinal tracts of these animals after oral gavage. Invasion was significantly delayed in mice carrying the parental *Bf*^N^ strain compared to mice carrying *Bf*^N^Δ*fab1* (Fig. S4A), although the relative abundance of the invading strain in mice carrying *Bf*^N^Δ*fab1* varied between 59% and 98% among individual mice by the end of the experiment. This interanimal variability was likely not due to genetic differences in these outbred animals, because repeating these studies in C57BL/6 (inbred) mice did not reduce the observed variability; indeed, the time points at which invading strain abundance was dependent on resident strain genotype differed in separate experiments (Fig. S4B and C). Laboratory-grown *Bf*^R^ exhibited minimal invasion in mice carrying *Bf*^N^ or *Bf*^N^Δ*fab1* (Fig. S4D). The *Bf*^N^Δ*fab1* mutant does not exhibit a fitness defect in direct competition with its isogenic parental strain in gnotobiotic mice, suggesting that a reduced capacity to delay invasion is not due to generic fitness defect in the gut (Fig. S5).

10.1128/mBio.00656-21.4 FIG S4 Impact of Fab1 on invasion of a susceptible strain in gnotobiotic mice. (A to C) Invasion of the *Bf*^R^ parental strain, harvested from monocolonized mice, into gnotobiotic mice carrying parental or Δ*fab1* mutant *Bf*^N^ strains. (A) Swiss-Webster (outbred) mice; (B to C), C57BL/6 (inbred) mice. (D) Invasion of the *Bf*^R^ parental strain, harvested from laboratory cultures, into gnotobiotic C57BL/6 mice carrying parental or Δ*fab1* mutant *Bf*^N^ strains. In panels A to D, the abundance of each strain was determined by quantitative PCR using gDNA from fecal samples collected over time. The relative abundance of the *Bf*^R^ parental strain in each group (*n* = 4 [A], 5 [B and C], and 10 [D]) is shown; error bars indicate SD. *, P < 0.05, **, P < 0.01, ***, P < 0.001, ****, P < 0.0001. Download FIG S4, EPS file, 2.2 MB. Copyright © 2021 Bao et al. 2021 Bao et al. https://creativecommons.org/licenses/by/4.0/ This content is distributed under the terms of the Creative Commons Attribution 4.0 International license.

10.1128/mBio.00656-21.5 FIG S5 A *fab1* deletion mutant does not have a fitness defect in gnotobiotic mice. *Bf*^N^ parental and Δ*fab1* mutant strains were introduced into C57BL/6 germfree mice (*n* = 5). The abundance of each strain was determined by quantitative PCR using gDNA from fecal samples collected over time. Error bars indicate SD. Download FIG S5, EPS file, 2.0 MB. Copyright © 2021 Bao et al. 2021 Bao et al. https://creativecommons.org/licenses/by/4.0/ This content is distributed under the terms of the Creative Commons Attribution 4.0 International license.

### Rfab1 protects susceptible strains from Fab1.

**FIG 5:**
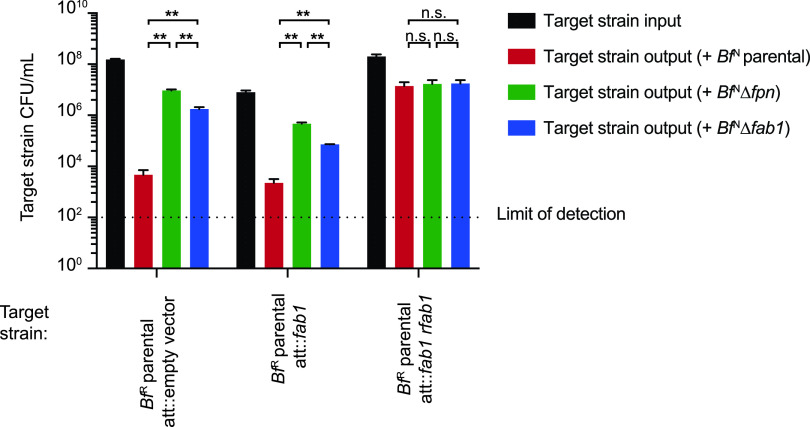
Expression of RFab1 protects otherwise susceptible strains from Fab1-mediated antagonism. Expression of the genetic cassette containing *fab1* and *rfab1*, but not *fab1* alone, in the susceptible *Bf*^R^ parental strain protects against Fab1-mediated antagonism. Input and output CFU of *Bf*^R^ target strains are reported. Error bars indicate SD (*n* = 2; representative of three independent experiments). **, *P < *0.01; n.s., not significant.

Antimicrobial toxins are frequently encoded in tandem with cognate immunity genes that protect against intoxication by sister cells (14, 19, 29). BF9343_1465 (Rfab1), encoded downstream of *fab1* (Fig. 2B), has no known function or recognizable domains. Notably, expression of the genetic fragment containing *fab1* and *rfab1* with their native promoter in the susceptible strain *Bf*^R^ protects this strain from Fpn- and Fab1-mediated killing (Fig. 5). Expression of *fab1* alone in *Bf*^R^ fails to confer any protective effect, implicating Rfab1 as an immunity factor. Accordingly, we designated this protein resistance to fragipain-activated bacteriocin 1 (Rfab1).

### The *fab1*/*rfab1* gene cluster is likely acquired through horizontal gene transfer independent of Fpn.

**FIG 6:**
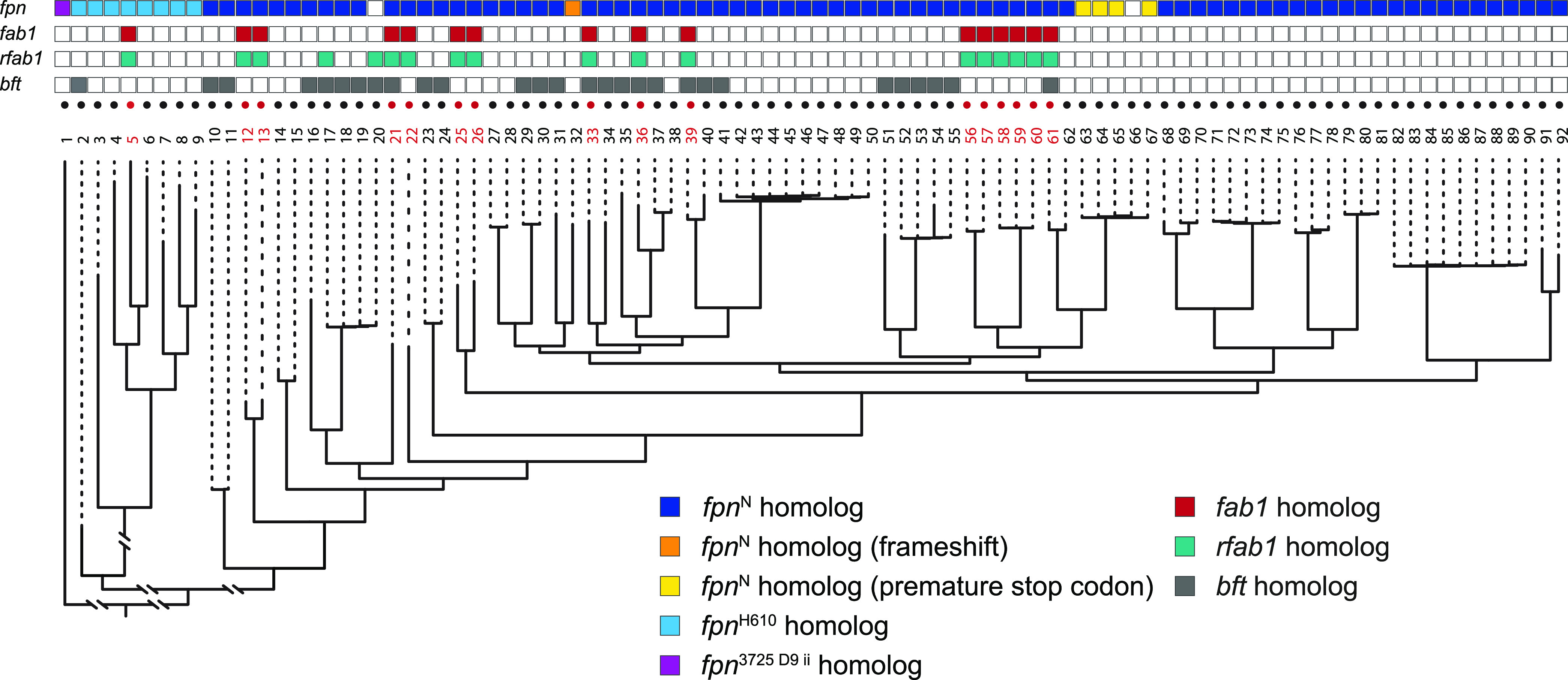
Comparative genomic analysis reveals multiple independent acquisitions of *fab1* and *rfab1* across B. fragilis strains. B. fragilis strains (numbered 1 to 92; Table S2) that carry a *fab1* homolog are in red, and strains with no identified *fab1* homolog are in black. (Bottom) Whole-genome phylogeny; (top) presence of *fpn* (purple, light blue, dark blue, orange, or yellow), *fab1* (red), *rfab1* (green), or *bft* (gray). Empty boxes indicate that no homolog was identified.

To assess the potential of diverse B. fragilis strains to utilize this antagonistic pathway, we first searched 92 sequenced B. fragilis genomes (14) for homologs of Fpn. Consistent with its distribution across prominent gut *Bacteroides* species (30), *fpn* is conserved in nearly all of these B. fragilis genomes (Fig. 6). These strains (including *Bf*^N^ and the ETBF strain B. fragilis ATCC 43859) generally encode two Fpn homologs, one sharing 70 to 100% identity and the other sharing 30% identity to *Bf*^N^ Fpn. The *fpn* allele encoded in *Bf*^H610^ (which has 73% amino acid sequence identity with *Bf*^N^ Fpn) restores antagonistic activity to a *Bf*^N^*Δfpn* mutant (Fig. 3C), suggesting that other homologs with 70 to 100% amino acid identity may share this capacity. Because deletion of *fpn* abolishes Fab1 activity in *Bf*^N^ (Fig. 2D) and BFT activity in *Bf* ATCC 43859 (25), the second (30% identity) homolog of Fpn likely targets substrates other than Fab1 and BFT (or is nonfunctional); as a result, we did not include it in our analysis.

10.1128/mBio.00656-21.7 TABLE S2 Ninety-two sequenced strains of B. fragilis analyzed in this study. Download Table S2, XLSX file, 0.01 MB. Copyright © 2021 Bao et al. 2021 Bao et al. https://creativecommons.org/licenses/by/4.0/ This content is distributed under the terms of the Creative Commons Attribution 4.0 International license.

Analysis of these genomes for *fab1* and *rfab1* homologs revealed that *fab1* is heterogeneously distributed within the B. fragilis phylogeny, consistent with repeated acquisition by horizontal gene transfer. *Fab1* is encoded in approximately 20% of analyzed B. fragilis genomes, and its presence is independent of the presence of *bft;* like *bft* and many B. fragilis T6SS-dependent effectors, *fab1* is rarely identified in genomes outside B. fragilis*. Rfab1* is encoded directly downstream of *fab1* in all *fab1*-positive genomes, consistent with a role as a cognate immunity factor for Fab1. Additionally, we identified two strains that encode *rfab1* without *fab*1, suggesting that Rfab1 may also function as an orphan immunity factor that protects these strains from Fab1-mediated killing by other strains. Together, these results suggest that the capacity of a strain to express or resist Fab1-mediated antagonistic activity will not be readily predicted by phylogeny.

## DISCUSSION

The gut microbiome harbors enormous bacterial populations (31). These microbes encode a diverse repertoire of contact-dependent and contact-independent mechanisms that determine strain fitness *in vitro* and in animal models (14, 19). Metagenomic analyses suggest that interbacterial antagonistic mechanisms can also provide a selective advantage in the human gut microbiome (6). However, how contact-independent antibacterial toxins are translocated and released from commensal microbes is not understood.

Human intestinal *Bacteroides*, specifically certain strains of B. fragilis, also translocate and secrete host-targeting toxins such as BFT, which causes epithelial cell damage in the cecum and colon (22). This predicted lipoprotein is exported to the cell surface; Fpn activity removes the N-terminal BFT prodomain to release the active toxin (26). Notably, ETBF B. fragilis strains also cause BFT-dependent host epithelial damage in the absence of Fpn (25). The observations that Fpn is dispensable for BFT activity in the gut and is encoded in many nontoxigenic strains are consistent with the hypothesis that this protease mediates other functions in human gut *Bacteroides*. Indeed, a recent secretome analysis of an ETBF strain and its isogenic Δ*fpn* mutant suggests that Fpn is likely involved in releasing many proteins in addition to BFT (26).

In this report, we demonstrate that in addition to its role in activating a host-targeting virulence factor, Fpn also mediates the activation and secretion of the potent antibacterial toxin Fab1. Several B. fragilis strains encode both BFT and Fab1 (Fig. 6), although whether these strains secrete both toxins under the same conditions is unknown. Cleavage by Fpn is important for both the secretion and activity of Fab1 (Fig. 4A and C). Purified Fab1 begins to aggregate after Fpn-mediated cleavage, consistent with a role for the prodomain in folding and stabilization. While the arginine residue at position 200 in Fab1 is required for normal cleavage and antibacterial activity of the protein (Fig. 4A to C), additional cleavage site(s) are likely because Fpn also alters the size of Fab1^R200A^ (Fig. 4B).

Fab1 does not contain any identifiable domains or high-confidence structural predictions, and its molecular target (and which species express this target) is unknown. Rfab1, which also does not contain any identifiable domains, is encoded directly downstream of *fab1* in all *fab1*-carrying strains. This conserved genetic organization and the observation that expression of a *fab1*-*rfab1* cassette (but not *fab1* alone) protects an otherwise susceptible strain from Fab1-mediated antagonism suggest that Rfab1 confers immunity against Fab1. Multiple B. fragilis strains that do not carry *fab1* do have *rfab1* homologs (98% identity with *Bf*^N^ Rfab1) in their genomes, suggesting that this gene has been repeatedly acquired and maintained as an orphan immunity factor to protect against antagonism by Fab1-producing strains. In two independent gnotobiotic experiments using inbred mice and a third experiment using outbred mice, Fab1 expression by *Bf*^N^ significantly delayed the invasion of the Fab1-sensitive strain *Bf*^R^ in the gut. However, the dynamics of the delay were variable between mice (and experiments) and sensitive to the origin of the invading strain. *Bf*^N^ and *Bf*^R^ vary by over 800 genes (32) and *Bf*^R^ exhibits a significant fitness advantage in competition with *Bf*^N^ *in vivo* (7); it is possible that Fab1 provides a local fitness advantage that is not readily measured in feces or primarily allows *Bf*^N^ to antagonize strains other than *Bf*^R^.

Notably, the *Bf*^N^Δ*fpn* deletion mutant exhibits significantly less antibacterial activity than the corresponding Δ*fab1* deletion mutant (Fig. 2D), suggesting that Fpn may be responsible for the activation of an additional antimicrobial factor(s) in *Bf*^N^. Because Fab1 lacks sequence similarity to any previously identified bacteriocins, a combination of genetic and proteomic methods may be required to identify additional Fpn-dependent bacteriocins.

Our study adds to the emerging evidence that diverse effector delivery pathways can deliver proteins that target both bacterial and host cells (14, 29, 33–35); selection for pathways that mediate antibacterial antagonism may prepare strains to utilize host-targeting toxins acquired by horizontal gene transfer, and vice versa. Future studies may also resolve whether bacteria- and host-targeting toxins provide a common benefit to the producer, possibly releasing nutrients from susceptible bacteria or host tissues or countering other antagonistic activities from the microbiome or host.

## MATERIALS AND METHODS

### Bacterial culture conditions.

*Bacteroides* strains were grown in liquid TYG (tryptone yeast glucose) medium, in liquid minimal medium (14), or on brain heart infusion (BHI; Becton Dickinson) agar supplemented with 50 mg/liter hemin (MP Biomedicals) and 0.5 mg/liter vitamin K_3_ (MP Biomedicals) in an anaerobic chamber (Coy Laboratory Products) filled with 70% N_2_, 20% CO_2_, and 10% H_2_. Escherichia coli S17-1 lambda *pir* and BL21 Rosetta strains were grown in LB medium and incubated aerobically at 37°C with shaking at 300 rpm. Antibiotics were added when required at the following concentrations: anhydrotetracycline (aTC), 2 ng/ml; ampicillin, 100 μg/ml; erythromycin, 25 μg/ml; gentamicin, 200 μg/ml; kanamycin, 50 μg/ml; tetracycline, 2 μg/ml; and 5-fluoro-2′-deoxyuridine (FUdR), 200 μg/ml.

### *Bacteroides* genetic manipulations.

All primers used in this study were obtained from the Keck Biotechnology Resource Laboratory (Yale University). DNA amplification was carried out using Kapa HiFi ReadyMix (Kapa Biosystems). The creation, maintenance, and transformation of plasmid constructs were performed using standard molecular cloning procedures. Primer sequences are provided in Table S1. The identity of the *Bf*^N^ and *Bf*^R^ strains used in these studies was confirmed by whole-genome sequencing and comparison to the reference genome sequences (GCA_900445515.1 and GCA_000210835.1).

**(i) Deletion of *tssC* from B. fragilis 638R.** The *Bf*^R^Δ*tssC* strain was constructed using pSIE2 (36). In brief, flanking regions (1,000 to 1,500 bp) of the *tssC* gene were PCR amplified (Table S1) and assembled with pSIE2 by Gibson cloning to make pSIE2-BF638R_tssC. This plasmid was sequence-verified and transformed into E. coli S17-1 λ*pir*, which was used for conjugation with *Bf*^R^. Merodiploids were selected by plating on BHI supplemented with gentamicin and erythromycin, and second recombination events were generated by overnight culture in TYG followed by plating on BHI supplemented with aTC as described previously (36). Individual clones were then screened by PCR for deletion of *tssC* (Table S1).

**(ii) Mutant construction in B. fragilis NCTC9343.***Bf*^N^Δ*tdk* and *Bf*^N^Δ*tdk*Δ*tssC* mutant strains were previously described (14), and all other *Bf*^N^ mutant strains were constructed using the *Bf*^N^*ΔtdkΔtssC* parental strain as previously described (37). Briefly, flanking regions (1,000 to 1,500 bp) of genes of interest were PCR amplified (Table S1) and assembled with pExchange-tdk (37) by Gibson assembly. The resulting vectors were sequence verified and cloned into E. coli S17-1 λ*pir* by transformation. Plasmids were then mobilized into *Bf*^N^Δ*tdk*Δ*tssC* by conjugation. Merodiploids were selected on BHI plates containing gentamicin and erythromycin, grown in liquid TYG to allow generation of second recombination events, and plated onto BHI agar supplemented with FUdR. Gene deletions were verified through PCR (Table S1).

**(iii) Genetic complementation.** Genes of interest were PCR amplified (Table S1), assembled with pNBU2 vectors (with or without oligonucleotide barcodes) by Gibson assembly, and introduced in single copy into *Bf*^N^ as previously described (38). Fpn and its mutants (Fpn^H135A^ and Fpn^C180A^) were cloned downstream of the synthetic promoter P5E4 (39). *fab1* and *rfab1* were cloned with the predicted endogenous promoter 300 bp upstream of *fab1*. Integration sites were verified through PCR (Table S1).

### Bacterial antagonism studies.

**(i) Transposon mutagenesis screen.** To create pSAM_BfN, pSAM_Bt (40) was modified by replacing the promoter upstream of the erythromycin resistance gene *ermG* with the promoter 300 bp upstream of the RpoD (BF9343_2673) gene of *Bf*^N^. The construct was verified by sequencing and transformed into E. coli S17-1 lambda *pir*. This strain was used for conjugation with *Bf*^N^ as described elsewhere (40). Clones with transposon insertions were selected on BHI agar with gentamicin and erythromycin and transferred into 96-well plates (termed *Bf*^N^::TN plates) containing TYG medium with erythromycin using a microbial colony picker (QPix 420; Molecular Devices, San Jose, CA, USA). After 20 h anaerobic incubation at 37°C, an aliquot from each well was individually combined with an equal volume of early-log-phase (optical density at 600 nm [OD_600_], 0.1) *Bf*^H610^ culture, and 10 μl of each mixture was spotted in a 96-well format onto nitrocellulose filters on BHI agar plates by robotic liquid handling (epMotion 5075; Eppendorf, Hamburg, Germany). Plates were incubated under anaerobic conditions at 37°C for 20 h, and spots were patched onto BHI agar with gentamicin and tetracycline to select for *Bf*^H610^. Transposon mutants from corresponding wells in the *Bf*^N^::TN plates were colony purified on BHI agar containing erythromycin and gentamicin. Colony-purified isolates were then subjected to secondary screening using the procedure described above and transposon insertion site mapping using primers described in Table S1 and a previously described protocol (40).

**(ii) Cocultivation assay.** The cocultivation assay was conducted largely as described elsewhere (14). Strains were grown on BHI agar plates for 16 to 20 h at 37°C. Bacterial lawns were resuspended from the plates in phosphate-buffered saline (PBS), and cell suspensions were adjusted to an OD_600_ of 0.1. Cells were mixed at a 1:1 (vol/vol) ratio, and 10 μl of each mixture was spotted onto nitrocellulose squares placed on BHI agar plates. After incubation at 37°C anaerobically for 8 h, viable cells were enumerated by serial dilution and selective plating based on the antibiotic resistance profile of each strain (Table S1). Significant differences were determined by repeated-measures analysis of variance (ANOVA) and *post hoc* Tukey’s honestly significant difference (HSD) test.

**(iii) Supernatant activity assay.***(a) Preparation of culture supernatants.* To minimize cellular material in supernatant activity assays, supernatant test cultures were initiated from actively growing starter cultures. To this end, supernatant producer strains were inoculated from glycerol stocks into 5 ml minimal medium and grown anaerobically at 37°C for 20 h. These cultures were subcultured (1:1,000), grown as described above to an OD_600_ of 0.6 to 0.8, and used to initiate test cultures at a starting OD_600_ of 0.02. These cultures were grown as described above to an OD_600_ of 0.3 before being pelleted by centrifugation at 3,220 × *g* for 10 min at 4°C. Supernatants were collected and filtered through a 0.2-μm filter. In certain studies, supernatants were heat treated at 95°C for 40 min or incubated with proteinase K (100 μg/ml) at 37°C for 30 min followed by 65°C for 10 min to inactivate proteinase K. In control samples, untreated supernatants were incubated at 25°C for 40 min.

*(b) Preparation of target strain cultures.* Target strains were inoculated from glycerol stocks into 5 ml minimal medium and grown anaerobically at 37°C for 20 h. After subculturing (1:1,000), cultures were grown to an OD_600_ of 0.15 to 0.3 and used to inoculate supernatants or medium controls (prepared as described above) at an OD_600_ of 0.001. Cultures were incubated at 37°C anaerobically and viable cells enumerated by serial dilution and plating. Significant differences were determined by repeated-measures ANOVA and *post hoc* Tukey’s HSD test.

**(iv) Protein activity assays.** For growth inhibition studies, target strains were inoculated from glycerol stocks into 5 ml TYG medium and grown anaerobically at 37°C for 20 h. After 1:1,000 dilution into fresh TYG medium, cultures were grown to an OD_600_ of 0.15 to 0.3 and used to inoculate TYG medium to a starting OD_600_ of 0.02. Cultures were supplemented with purified proteins at 20 μg/ml (for controls containing Fpn or Fab1 alone) or Fab1 at 15 μg/ml and Fpn at 5 μg/ml (for test cultures containing both proteins).

For MIC_50_ determination, the *Bf*^R^ target strain was inoculated from glycerol stocks into 5 ml TYG medium and grown anaerobically at 37°C for 20 h. After 1:250 dilution into fresh TYG medium, cultures were grown to an OD_600_ of 0.2 to 0.3, and a bacterial pellet corresponding to 1 ml of culture was resuspended in 1× PBS to a final concentration of ∼10^5^ CFU/ml. Recombinant Fab1 was added at final concentrations of 0, 0.25, 0.5, 1, and 2 μg/ml, and Fpn was added at a final concentration of 2 μg/ml at all Fab1 concentrations. The cells were incubated with Fpn and Fab1 at 37°C, anaerobically, for 2 h before plating dilutions on BHI-HK (BHI-hemin-vitamin K) agar to determine CFU/ml.

### Protein and molecular assays.

**(i) Reverse transcription-quantitative PCR.** Cells were harvested at an OD_600_ of 0.3 with RNA Protect (Qiagen) according to the manufacturer’s instructions. RNA was extracted using the RNeasy kit (Qiagen) and reverse transcribed into cDNA using SuperScript II reverse transcriptase (Thermo Fisher Scientific) with random priming. Quantitative PCR (qPCR) was performed using a CFX96 detection system (Bio-Rad) and SYBR FAST universal master mix (Kapa Biosystems) with primers described in Table S1. Primers were optimized using an Integrated DNA Technologies (IDT) OligoAnalyzer such that primers used together have melting temperatures within ∼2°C. Expression levels were compared by normalizing *fpn* and *BF9343_2672* transcripts to the amount of 16S transcript present in each sample.

**(ii) Expression and purification of bacterial proteins.**E. coli BL21 Rosetta carrying pET21_NESG expression vectors (38) were used for protein expression and purification. BFT was amplified using published primer sequences (25) and assembled with pET21_NESG using Gibson assembly. For both Fpn and Fab1, N-terminal predicted signal sequences were omitted from the open reading frames and a C-terminal 6×His tag and/or C-terminal c-Myc-tag was added and cloned into pET21_NESG (Table S1).

For protein purification, E. coli expression strains were grown for 20 h, subcultured (1:200), and allowed to grow to an OD_600_ of 0.4 to 0.6, before induction with IPTG (isopropyl-β-d-thiogalactopyranoside; 0.5 mM) for 4 h. Cells were harvested and lysed using BugBuster reagent (Millipore Sigma, Burlington, MA, USA). Lysates were incubated for 1 h at 4°C with nickel-nitrilotriacetic acid (Ni-NTA) agarose beads (Qiagen, Hilden, Germany), washed with 30 ml of wash buffer (50 mM NaH_2_PO_4_, 300 mM NaCl, 20 mM imidazole [pH 7.4]), and eluted with 5 ml elution buffer (50 mM NaH_2_PO_4_, 300 mM NaCl, 250 mM imidazole [pH 7.4]). Fab1 was further purified using a Pierce strong cation exchange column (Thermo Fisher Scientific, Waltham, MA, USA).

**(iii) *In vitro* cleavage of BFT and Fab1.** Purified proteins (BFT, Fab1, and Fpn) were dialyzed twice against PBS. Equimolar amounts of wild-type Fpn or mutant variants were incubated with substrates in PBS at 37°C for 30 min followed by deactivation at 95°C for 5 min.

**(iv) Immunoblotting.** Proteins were separated by SDS-PAGE and transferred onto a polyvinylidene difluoride (PVDF) membrane (Bio-Rad). Membranes were blocked with 5% nonfat milk in PBS with 0.1% Tween 20 (PBST). Primary antibodies, including anti-C-myc mouse monoclonal antibody (Invitrogen) and anti-His_6_ mouse monoclonal antibody (Invitrogen), were diluted in 5% nonfat milk in PBST at 1:1,000; secondary antibodies were diluted in PBST at 1:10,000.

### Gnotobiotic animal studies.

All animal experiments were performed using protocols approved by the Yale University Institutional Animal Care and Use Committee. Male and female germfree 10- to 16-week-old Swiss Webster or C57BL/6 mice were individually caged and maintained in flexible plastic gnotobiotic isolators with a 12-h light/dark cycle. Mice were provided with standard autoclaved mouse chow (5K67 LabDiet; Purina, St. Louis, MO, USA) *ad libitum*.

**(i) *Bf*^N^ competition.***Bf*^N^ parental and *Bf*^N^Δ*fab1* strains were stored as single-use aliquots in 10% glycerol at −80°C. After CFU quantification from representative aliquots of each strain, strains were thawed, mixed, and introduced into germfree mice by oral gavage at a starting ratio of 1:1 (5 × 10^8^ CFU:5 × 10^8^ CFU).

**(ii) *Bf*^R^ invasion experiments.** Invasion experiments were conducted using two previously described protocols (3, 7). In the first approach, germfree mice were monoassociated (day −7) with 10^9^ CFU of either barcoded parental *Bf*^N^ (*n* = 5) or barcoded *Bf*^N^Δ*fab1* (*n* = 5) by oral gavage. One mouse was monoassociated with 10^9^ CFU of the barcoded *Bf*^R^ parental strain. On day −3, a fecal sample was collected from the *Bf*^R^ monoassociated mouse, and viable cells were enumerated by serial dilution and plating. On day 0, *Bf*^N^-monoassociated mice were subjected to gavage with fecal material from the *Bf*^R^-monoassociated mouse (10^8^ CFU, determined based on the CFU assessment from day −3). In the second approach, germfree mice were monoassociated (day −7) with 10^9^ CFU of either barcoded parental *Bf*^N^ (*n* = 5) or barcoded *Bf*^N^Δ*fab1* (*n* = 5) by oral gavage. Stationary-phase *Bf*^R^ cultures were resuspended in PBS with 20% glycerol and stored in aliquots at −80°C prior to CFU determination. On day 0, *Bf*^N^-monoassociated mice were subjected to gavage with 10^8^ CFU of these *in vitro*-grown *Bf*^R^ cultures. For all mouse experiments, fecal samples were collected over time and stored at −20°C before genomic DNA extraction. Total gDNA was extracted, and the relative abundance of each strain was determined by qPCR using a CFX96 detection system (Bio-Rad), SYBR FAST universal master mix (Kapa Biosystems), and oligonucleotide barcode-specific primers (Table S1) (41). Significant (*P < *0.05) differences were determined by Student's *t* test.

### Genomic analysis.

**(i) Genome phylogeny.** The phylogenetic tree of B. fragilis strains (listed in Table S2) is adapted from a previous study (14). In that study, these genomes (many of which are in draft stage) were also queried for the presence of 14 housekeeping genes conserved across all bacteria; for each genome, 14/14 of these genes were successfully identified (14).

Protein homolog identifications were conducted through BLASTp using the PATRIC database (42) as described below.

**(ii) Identification of *fpn* homologs.** The presence/absence of *fpn* was identified by BLASTp search using an E-value cutoff of 9e−43. *fpn* homologs were manually categorized into groups based on reciprocal BLAST using an identity cutoff of 75% and screened for mutations.

**(iii) Identification of *bft* homologs.** The presence/absence of *bft* was identified by BLASTp search for the 186-residue active fragment (43) of translated *bft* from strain ATCC 43859 using an E-value cutoff of 2e−35 and identity cutoff of 87%.

**(iv) Identification of *fab1* homologs.** The presence/absence of *fab1* was identified by BLASTp search using an E-value cutoff of 0.0 and identity cutoff of 60%.

**(v) Identification of rfab1 homologs.** The presence/absence of *rfab1* was identified by BLASTp search using an E-value cutoff of 4e−18 and identity cutoff of 95%.
